# Supplementary material for: Nutritional Interventions with Bacillus coagulans Improved Glucose Metabolism and Hyperinsulinemia in Mice with Acute Intermittent Porphyria
Source: Int J Mol Sci. 2023 Jul 26;24(15):11938. doi: 10.3390/ijms241511938 (PMC10418637; doi:10.3390/ijms241511938)

Figure S1

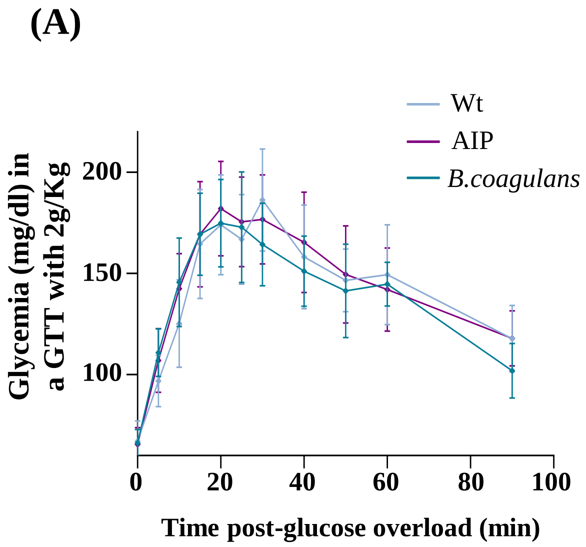
(A)
Wt
AIP
B.coagulans
Glycemia (mg/dl) in a GTT with 2g/Kg
200
150
100
0
20
40
60
80
100
Time post-glucose overload (min)

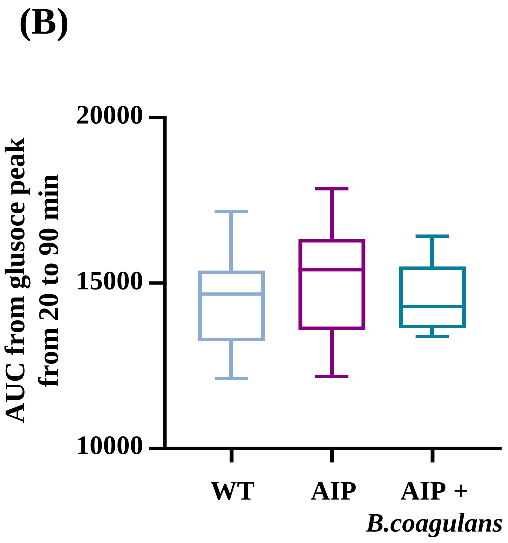
(B)
AUC from glusoce peak from 20 to 90 min
20000
15000
10000
WT
AIP
AIP + B.coagulans

Figure S2

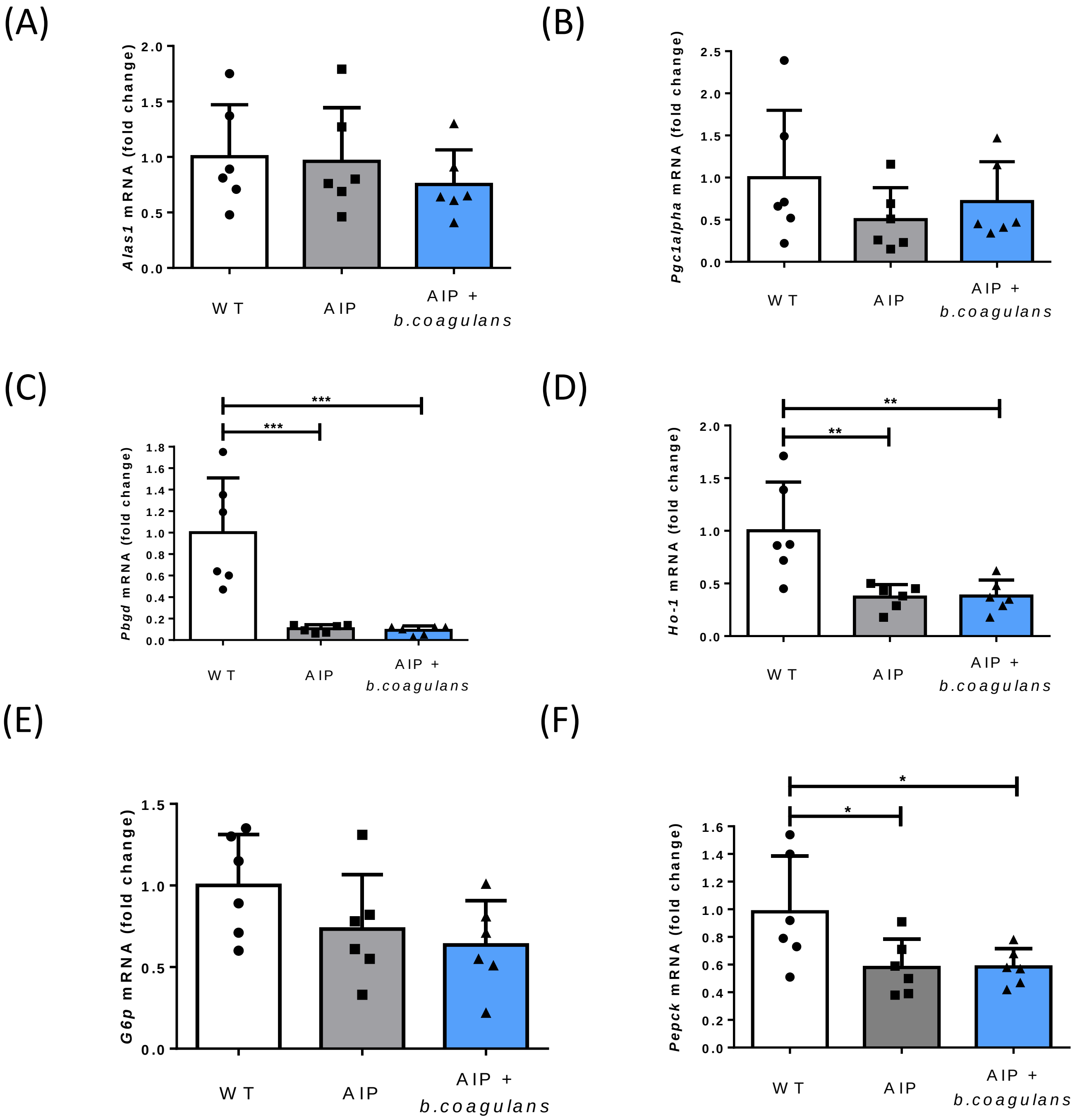

(A)
Alas1 mRNA (fold change)
2.0
1.5
1.0
0.5
0.0
WT
AIP
AIP +
b.coagulans
(B)
Pgc1alpha mRNA (fold change)
2.5
2.0
1.5
1.0
0.5
0.0
WT
AIP
AIP +
b.coagulans
(C)
***
***
Pbgd mRNA (fold change)
1.8
1.6
1.4
1.2
1.0
0.8
0.6
0.4
0.2
0.0
WT
AIP
AIP +
b.coagulans
(D)
**
**
Ho-1 mRNA (fold change)
2.0
1.5
1.0
0.5
0.0
WT
AIP
AIP +
b.coagulans
(E)
G6p mRNA (fold change)
1.5
1.0
0.5
0.0
WT
AIP
AIP +
b.coagulans
(F)
*
*
Pepck mRNA (fold change)
1.6
1.4
1.2
1.0
0.8
0.6
0.4
0.2
0.0
WT
AIP
AIP +
b.coagulans

Figure S3

(A)

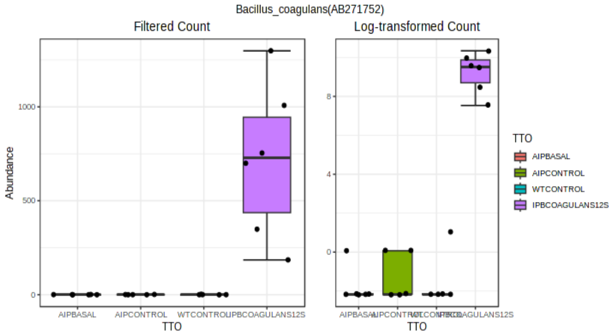

(B) Alfa diversity (Observed, P=0.003)

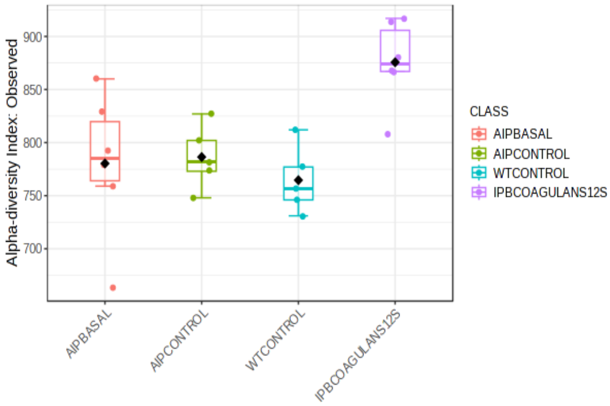

(C) Beta diversity :

(Feature-level PCoA Bray-Curtis, P=0.001)

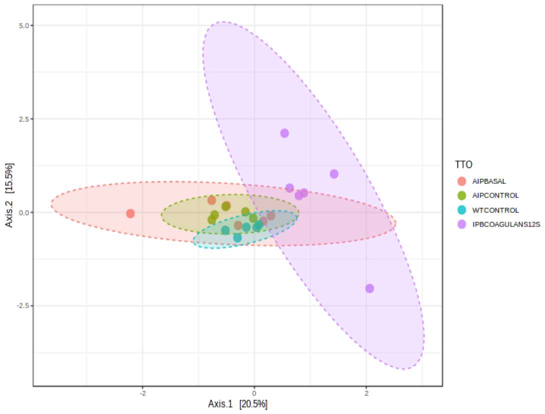

Supplement: Supplementary file 1 [file ijms-24-11938-s001.zip › ijms-2505865-supplementary.pdf]
